# The Presence of Virus Neutralizing Antibodies Is Highly Associated with Protection against Virulent Challenge in Domestic Pigs Immunized with ASFV live Attenuated Vaccine Candidates

**DOI:** 10.3390/pathogens11111311

**Published:** 2022-11-08

**Authors:** Ediane B. Silva, Peter W. Krug, Elizabeth Ramirez-Medina, Alyssa Valladares, Ayushi Rai, Nallely Espinoza, Douglas P. Gladue, Manuel V. Borca

**Affiliations:** 1 Plum Island Animal Disease Center, ARS, USDA, Greenport, NY 11944, USA; 2 College of Veterinary Medicine, Kansas State University, Manhattan, KS 66506, USA; 3 Oak Ridge Institute for Science and Education (ORISE), Oak Ridge, TN 37830, USA

**Keywords:** ASFV, ASF, African swine fever virus, virus neutralizing antibodies, protective immunity

## Abstract

African swine fever virus (ASFV) is currently producing a pandemic affecting a large area of Eurasia, and more recently, the Dominican Republic in the Western Hemisphere. ASFV is a large and structurally complex virus with a large dsDNA genome encoding for more than 150 genes. Live attenuated virus strains can induce protection in domestic swine against disease produced by homologous virulent parental viruses. The roles of the different immune mechanisms induced by the attenuated strains in protection still need to be understood. In particular, the role of ASFV neutralizing antibody in protection still is an important controversial issue to be elucidated. Here we present the development of a novel methodology to detect virus neutralizing antibodies based on the reduction of virus infectivity in a Vero cell adapted ASFV strain. The described method was used to assess levels of virus neutralizing antibodies in domestic swine inoculated with live attenuated ASFV. Results demonstrated a high association between the presence of virus neutralizing antibodies and protection in 84 animals immunized with the recombinant vaccine candidates ASFV-G-Δ9GL/ΔUK or ASFV-G-ΔI177L. To our knowledge, this is the first report demonstrating an association between virus neutralizing antibodies and protection against virulent challenge in such a large number of experimental individuals.

## 1. Introduction

African swine fever (ASF) is a usually lethal disease of domestic pigs which is currently producing a pandemic affecting the swine production industry across Eurasia and just recently, the Hispaniola Island, after more than 40 years of being absent in the Western Hemisphere [1]. Since the first available commercial vaccine is still restricted in its use [2,3], the current control of the disease is based on culling all infected animals along with strict measures to avoid the mobilization of susceptible and potentially infected animals. 

Protection against the disease has been experimentally demonstrated by immunizing animals with live attenuated strains of viruses. Live attenuated strains, particularly those produced by the deletion of specific genes involved in virulence from the genome of virulent field ASFV isolates, are generally effective at inducing protection against the challenge with the homologous virulent strains [4,5,6,7,8]. Nevertheless, the host immune mechanism mediating the protection induced by those live attenuated strains is far from being identified. Although several immune mechanisms have been described as associated with ASF infection [9,10], their role in the induction of protection has not been conclusively demonstrated. There is evidence that the T-cell immunity is important in the protective immune response elicited by live attenuated vaccine candidates [11,12]. Similarly, passive transfer of immunoglobulin from ASF convalescent animals protected a high proportion of recipient naïve animals against challenge with virulent virus [13]. Nevertheless, the immune effector mechanism mediated by the ASF specific antibodies remains to be elucidated. Several laboratories have developed different methodologies to detect the presence of ASFV neutralizing antibodies [14,15,16,17,18]. However, the potential role of virus neutralizing antibodies in protection against infection and disease after challenge with virulent virus strains remains uncertain. In this report, we attempt to associate the presence of virus neutralizing antibodies and protection using a reliable experimental model involving domestic pigs immunized with protective ASFV recombinant vaccine candidates followed by virulent challenge. By assessing the sera from over 80 experimentally vaccinated animals, our results demonstrated a close association between the presence of virus neutralizing antibodies and protection against lethal ASFV challenge

## 2. Materials and Methods

### 2.1. Preparation of Virus Neutralization Stock 

Vero cells (CCL-81, ATCC) were passaged using growth media consisting of DMEM supplemented with 7% fetal bovine sera (FBS) and 1X antibiotics. High-titer Vero cell-adapted ASFV from the 30^th^ passage (ASFV/VP30) stocks [19] were diluted tenfold in maintenance media (DMEM supplemented with 1% FBS and 1X antibiotics), then Tween 80 was added to a final concentration of 0.05%. After extensive mixing, the stock was sonicated on ice 3 times for 15 s each with a microtip sonicator (Branson Ultrasonics™ Sonifier™ Cell Disruptor) set at 30% power to prevent virion aggregates. The virus stock was then filtered on 0.45µm syringe filters, aliquoted and stored at −70 °C until use. To determine virus titer, serial dilutions of this stock were used to infect Vero cells on 12-well cell culture plates. One hour after infection, the inoculum was removed, and the wells were overlayed with methylcellulose media (0.5% methylcellulose in DMEM supplemented with 1% FBS and 1X Penicillin-streptomycin). Ten days post infection, the methylcellulose media was aspirated, the cells were washed with PBS, fixed with a 1:1 mixture of methanol:acetone and the plaques were visualized by immunostaining using a monoclonal antibody recognizing ASFV protein p30 as previously described [20]. Virus stocks that contained at least 1 × 10^7^ PFU/mL were used for the neutralization assay.

### 2.2. Neutralization Assay 

This assay is a modification of the procedure originally described by Zsak et al. [18]. Heat-inactivated swine sera was serially diluted tenfold with maintenance media in non-tissue culture-treated 96-well dilution plates. An equal volume containing 100 PFU of ASFV/VP30 neutralization stock was added to each well of diluted serum, and the plates were incubated at 37 °C in a cell culture incubator with 5% CO_2_ for 18–24 h. Wells containing 100 PFU of virus mixed with naïve swine serum were included as controls. Twelve-well cell culture plates containing 90% confluent Vero cell monolayers were washed once with phosphate-buffered saline (GIBCO^TM^) to remove growth media and then infected with the serum:virus neutralization mixtures at 37 °C in a 5% CO_2_ cell culture incubator. After one hour incubation, the inoculum was removed, and methylcellulose media was added to each well. Plates were then placed in a 37 °C cell culture incubator with 5% CO_2_ for up to 10 days. After confirming the naïve serum control wells exhibited visible virus plaques by light microscopy, the methylcellulose overlay was removed from all the wells by aspiration and the wells were washed once with PBS to remove the remaining methylcellulose media. The wells were fixed with a 1:1 mixture of ice-cold methanol:acetone for 15 min and after the fixative was removed, plates were allowed to dry. Presence of plaques were visualized by immunostaining performed as described elsewhere [20]. The number of plaques on each well was determined by counting under a dissecting microscope. The number of plaques for each serum dilution was plotted against each serum concentration and the trendline equation was generated using Microsoft Excel. The neutralization index (NI) is defined as the dilution representing the serum concentration for each sample that inibits 50% of the viral plaques counted in the naïve serum control wells.

### 2.3. Detection of ASFV Specific Antibody Response by ELISA 

Detection of ASFV specific antibody was performed using an in-house ELISA as described previously [20]. Briefly, ELISA antigen was prepared from Vero cells infected with a Vero adapted Georgia strain ASFV. Maxisorb ELISA plates (Nunc, St. Louis, MO, USA) were coated with 1 µg per well of infected or uninfected cell extract. The plates were blocked with phosphate buffered saline containing 10% skim milk (Merck, Kenilworth, NJ, USA) and 5% normal goat serum (Sigma, Saint Louis, MO, USA). Each swine serum sample was tested at multiple dilutions against both infected and uninfected cellular antigen. ASFV-specific antibodies in the swine sera were detected using an anti-swine IgG-horseradish peroxidase conjugate (KPL, Gaithersburg, MD, USA) and SureBlue Reserve peroxidase substrate (KPL). Plates were read at OD630 nm in an ELx808 plate reader (BioTek, Shoreline, WA, USA). Antibody titers were expressed as the log_10_ of the inverse highest dilution where the OD630 nm reading of the tested sera at least duplicated the reading of the mock infected (obtained at day 0 post infection) sera.

### 2.4. Animal Study Design

In order to analyze the potential association of the presence of serum neutralizing antibodies and protection against disease, a set of sera from animals that were vaccinated with experimental recombinant live attenuated vaccines and further challenged with the virulent parental field strain were selected. A total of 84 sera obtained at the time of challenge from pigs vaccinated with experimental vaccine candidates ASFV-G-Δ9GL/ΔUK [4] and ASFV-G-ΔI177L [8] were analyzed for neutralizing antibodies. The serum samples used in this study had been collected in previous studies [4,8,21] run under protocol approved by the Plum Island Animal Disease Center Institutional Animal Care and Use Committee (225.01-16-R_090716). These animals were vaccinated using different vaccines; doses, routes of inoculation and the challenge were conducted at different times post vaccination as described in each of the corresponding figures and it is summarized in Table 1. This set of sera contains sample form animals selected to represent a variety of scenarios regarding the protective response induced by intramuscular (IM) or oronasal (ON) vaccination and considering a different level of maturation of the antibody response. 

### 2.5. Statistical Analysis 

Data were analyzed using Microsoft Excel 2010 software and GraphPad prism 7 software. Comparison of the treated vaccine groups was analyzed by ANOVA with Dunnett’s multiple comparison test. Correlations between animal survival and sera virus neutralization capability and antibody specificity and virus neutralization were analyzed using Pearson correlation coefficients. The receiver operating characteristic (ROC) curve was analyzed to select optimal cutoff for virus neutralization value.

## 3. Results and Discussion

### 3.1. Association between the Presence of ASFV Specific Antibodies Detected by ELISA and Protection against the Virulent Challenge in Pigs Immunized with Live Attenuated Vaccine Candidates

**Figure 1:**
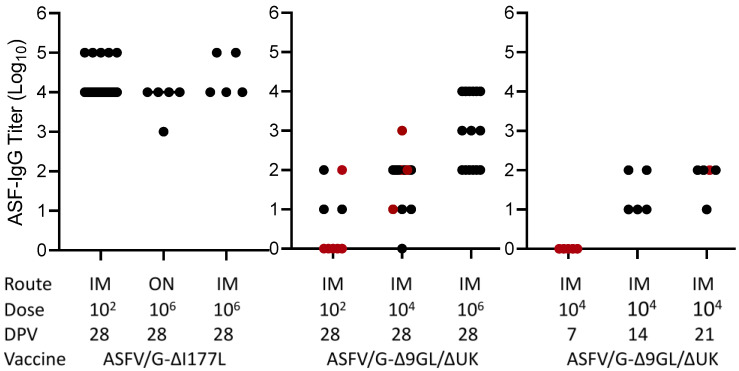
ELISA titers in sera obtained at the time of challenge in animals immunized with either ASFV-G-Δ9GL/ΔUK or ASFV-G-ΔI177L vaccine stains. Black dots and red dots indicate presence and absence of protection against the challenge, respectively.

First, the presence of ASFV specific antibodies in the set of the 84 sera was evaluated by the ELISA as described in Materials and Methods. Results indicated that most vaccinated animals possess levels of virus specific binding antibodies at the time of challenge (Figure 1). The exceptions were a subset of animals vaccinated with ASFV-G-Δ9GL/ΔUK either receiving 10^2^ HAD and challenged at 28 dpv or those receiving 10^4^ HAD and challenged at 7 dpv. As expected, titer values increased in groups of animals challenged at 28 dpv compared to earlier challenge at 7, 14 and 21 dpv (*p* < 0.0001, <0.0001, 0.0003). Detectable antibody titer over 10^1^ is generally associated with protection against clinical disease after challenge (Figure 1) with the exception of five animals immunized with ASFV-G-Δ9GL/ΔUK (one vaccinated with 10^2^ HAD and challenged at 28 dpv, and four vaccinated with 10^4^ HAD that were challenged at 21 and 28 dpv). Conversely, only four animals inoculated with ASFV-G-Δ9GL/ΔUK were protected at challenge without showing virus specific antibody titers, both vaccinated with 10^4^ HAD and challenged at 7 and 28 dpv, respectively. Therefore, out of the 84 cases reviewed, only 5 animals were not protected in the presence of antibodies and only 2 were protected in the absence of them. These results are in agreement with our previous observation that the presence of medium to high ASFV specific antibody titers at the time of challenge closely associates with protection against clinical disease [20].

### 3.2. Detection of Virus Neutralizing Antibodies in Pigs Immunized with Live Attenuated Vaccine Candidates

**Figure 2:**
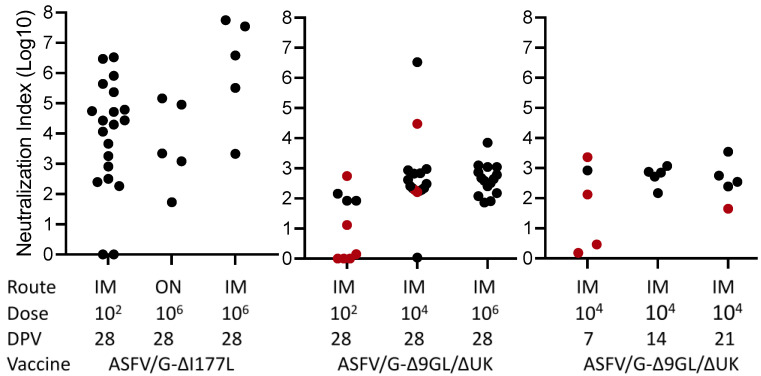
Neutralization index in sera obtained at the time of challenge in animals immunized with either ASFV-G-Δ9GL/ΔUK or ASFV-G-ΔI177L vaccine stains. The ROC curve was calculated with AUC = 0.7745 and Cut-off < 1.690 with specificity of 95.71% and Sensitivity of 50%. Black dots and red dots indicate presence and absence of protection against the challenge, respectively.

Next, to evaluate the relationship of virus neutralizing antibodies with ASF protection, the virus neutralization activity of the 84 sera from the pigs vaccinated with the two different recombinant virus strains was assessed (Figure 2). All animals vaccinated with ASFV-G-ΔI177L survived the challenge and exhibited NI values over 1.5, with the exception of two animals that were immunized with 10^2^ HAD_50_ that did not show any neutralizing activity. As observed with the antibodies detected by ELISA, overall, animals vaccinated with the ASFV-G-ΔI177L strain had a higher neutralization index (NI) compared with those vaccinated with ASFV-G-Δ9GL/ΔUK (*p* < 0.05). This is particularly evident (*p* = 0.0004) when groups immunized with low doses of vaccine (10^2^ HAD_50_) and challenged at 28 dpv are compared: all animals receiving ASFV-G-ΔI177L developed NI over 2 while those immunized with group ASFV-G-Δ9GL/ΔUK barely reach that value. Considering the animals vaccinated with ASFV-G-Δ9GL/ΔUK, all of them demonstrated NI values over 1.5 and were protected against challenge with the exception of one receiving 10^2^ HAD_50_ and six receiving 10^4^ HAD_50_, challenged at 7, 21 and 28 dpv. Therefore, out of the 84 cases considered in the study, only 7 pigs with NI values over 1.5 were not protected against the challenge. Interesting, only one animal, vaccinated with 10^4^ HAD_50_ of ASFV-G-Δ9GL/ΔUK and challenged at 28 dpv, was protected without generating detectable neutralizing antibodies. Thus, in over 90% of the animals there existed an association between the presence of neutralizing antibody and protection against challenge.

**Figure 3:**
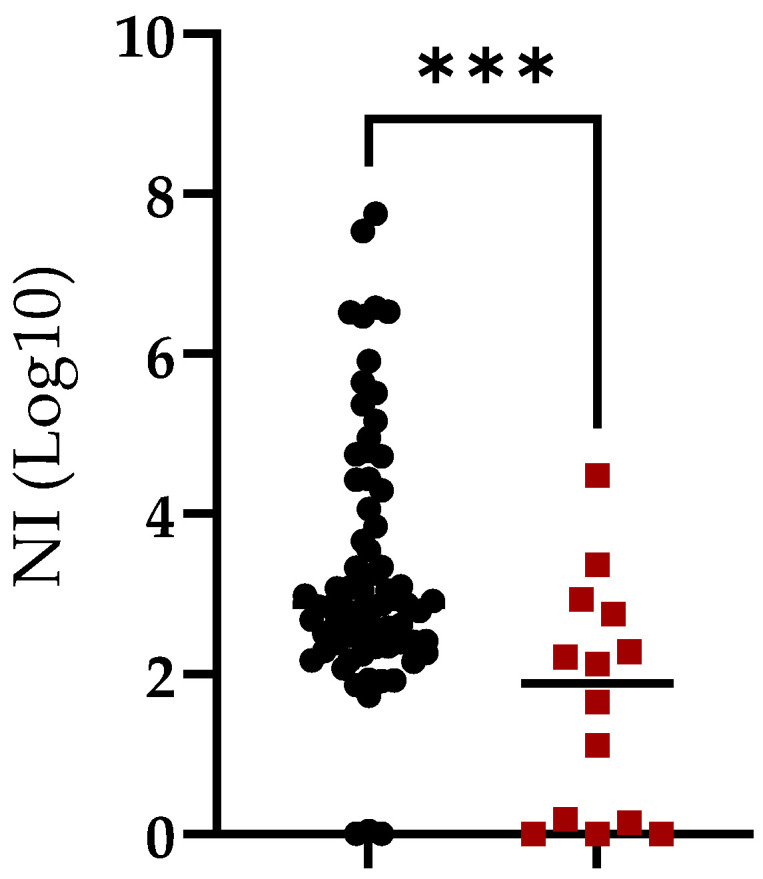
Neutralization index (NI) in sera of animals vaccinated with ASFV-G-Δ9GL/ΔUK or ASFV-G-ΔI177L vaccine strains and challenged with virulent field strain ASFV-G. Animals were grouped based on survival (black symbols) or absence of protection (red symbols) after the challenge. Asterisks indicate differences among groups (*p* < 0.05).

In the group of animals surviving the challenge, 95.7% of them demonstrated NI activity and only 3 out 70 (4.3%) failed to induce neutralizing antibodies against ASFV. Therefore, there was a strong association between survival after the challenge and NI activity. Our study, confirmed by ELISA (97.6%) and NI activity (95.7%) (Figure 3), suggests that survival from ASFV challenge is associated with the presence of antibodies.

**Figure 4:**
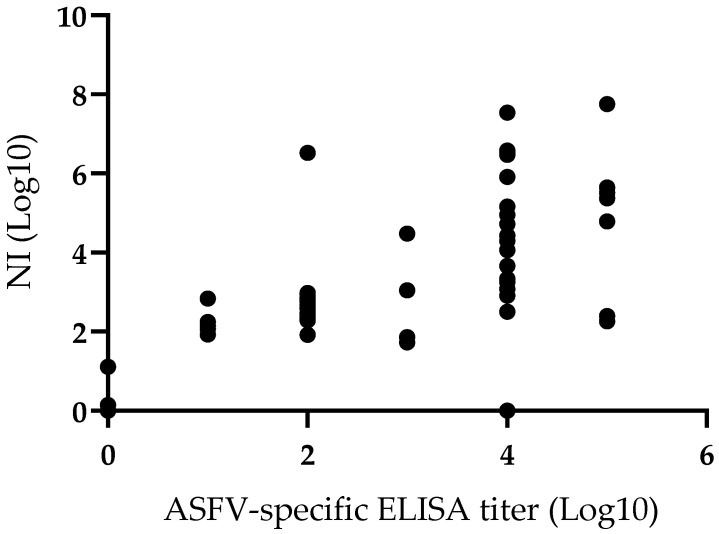
Comparison of neutralization index (NI) and anti-ASFV ELISA titers in sera of animals vaccinated with ASFV-G-Δ9GL/ΔUK or ASFV-G-ΔI177L vaccine stains. Pearson correlation coefficient (r) 0.65; 95% of interval 0.5004 to 0.7898; P (two-tailed) 0.0001.

In addition, the correlation between the presence of ASFV specific antibodies detected by ELISA and those detected by virus neutralization was analyzed. A Pearson correlation coefficient was calculated to establish this potential association and results indicate the presence of a significant positive correlation (r = 0.65; *p* = 0.0001) between the two variables considered (Figure 4). Regardless of the characteristics and inherent limitations of both techniques, the correlation related to the presence of antibodies detected by either methodology is clear. This correlation is not unexpected since it is reasonable to assume that the majority of antibody mediating virus neutralization will also be detected by the ELISA test used here. Consequently, the presence of antibodies detected by both techniques is consistently associated with protection against the challenge.

**Figure 5:**
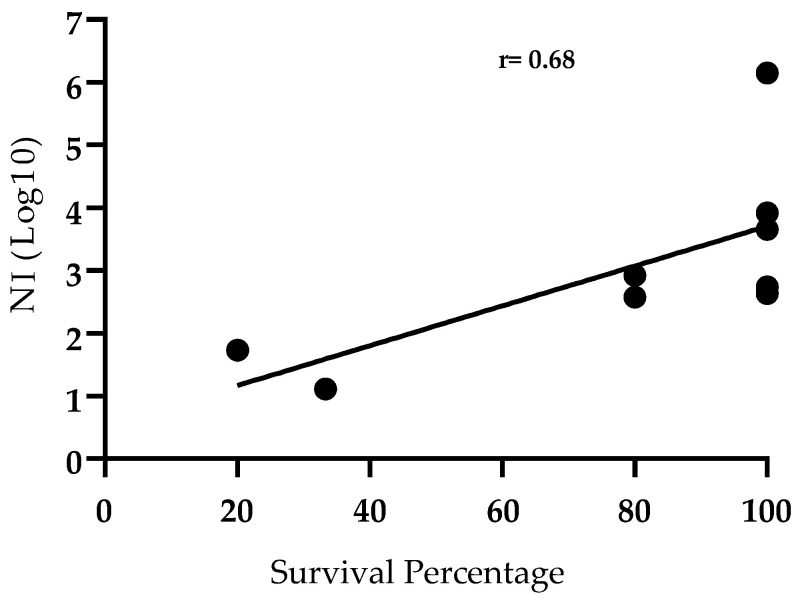
Correlation between protection after challenge and Neutralizing Index (NI) in sera of animals vaccinated with ASFV-G-Δ9GL/ΔUK or ASFV-G-ΔI177L vaccine stains. Survival percentage is represented by number of survivals groups described in Table 1. Pearson correlation coefficient r = 0.6841, *p* < 0.05; 95% confidence interval 0.0368 to 0.9271; P (two-tailed) 0.0421.

In summary, out of the 84 animals considered in this study 70 of them were protected against the challenge while the other 14 of them succumbed to lethal infection. In the group of animals surviving the challenge, 95.7% of them presented virus neutralizing antibody while, on the other hand, only 3 out 70 (4.3%) of those animals failed to produce detectable neutralizing antibodies against ASFV. Therefore, this suggests an association between animal survival at the time of challenge and VN activity. The Pearson correlation between survival and antibody neutralizing activity further demonstrates this relationship (Figure 5). There was a positive correlation between the two variables (r = 0.68; *p* = 0.0421). 

The neutralization assay developed here was based on a protocol designed to test the neutralizing capacity of monoclonal antibodies and swine serum to block ASFV infection as described in Zsak et al. [18]. The major difference between the assays is the use of a Vero cell-adapted Georgia strain virus at the 30^th^ passage, which is representative of the virus circulating in the current Eurasian pandemic [19]. Another difference is based on the readout of the assay. While the previous work [18] determined a percentage reduction for each serum or antibody tested at a predetermined dilution, the assay described here uses multiple dilutions for each serum to determine a 50% inhibition titer. Since there is always a fraction of the virus that is not neutralizable, the calculations were carried out with multiple serum dilution controls for this fraction. Using the average neutralization index among each group of animals given various vaccines at different doses, we were able to correlate high survival percentage after virulent challenge with high neutralizing antibody levels using a non-linear curve fit analysis, suggesting that neutralizing antibodies are a correlate of protection. Potential modifications of this assay would include high-throughput sample processing to analyze neutralizing antibodies using fluorescently labeled ASFV, enabling neutralization index analysis to become part of the pre-challenge quality control for vaccine candidates prior to challenge.

The presence of neutralizing antibodies in ASFV has been an issue of historical controversy among research groups working in the identification of host immune mechanisms mediating protection against virulent challenge. Although early findings supported the absence of virus neutralizing antibodies in ASFV [22,23] (perhaps due to assay limitations), numerous reports have shown the existence and characterization of neutralizing antibodies in the sera of animals surviving the infection with attenuated viruses (reviewed in [14]). It is now widely accepted that antibodies in animals surviving infection or those that have been treated with vaccine candidates have activity-blocking virus infectivity. The mechanism of this inhibition may not necessarily be virus neutralization per se, as protective antibodies can function in other ways (e.g., antibody-dependent cellular cytotoxicity, opsonization, complement-mediated lysis). Additionally, antibodies may work in cooperation with T-cell-mediated mechanisms of protection [24]. The results presented here indicate that the presence of ASFV neutralizing antibodies is associated with the protection against clinical disease and death produced by virulent challenge. In this report, we show that virus neutralization activity was present in almost 100% of animals vaccinated with live attenuated vaccine candidates that survived to the ASV challenge. This study attempted, for the first time, to associate the presence of ASFV neutralizing antibodies with protection from challenge utilizing a large number of experimentally vaccinated animals. The presented results suggest that antibody plays an important role in ASF protection. 

## Figures and Tables

**Table 1:** Detail of the vaccine type, route of administration, dose, time of challenge and protection status of pigs’ sera used in this report.

Vaccine Type, Dose, and Route of Administration *	Challenge at dpv ^†^	Number of Protected/ Total at Challenge ^††^
ASFV-G-Δ9GL/ΔUK (IM) 10^2^ HAD	28	3/9
ASFV-G-Δ9GL/ΔUK (IM) 10^4^ HAD	28	13/15
ASFV-G-Δ9GL/ΔUK (IM) 10^6^ HAD	28	15/15
ASFV-G-Δ9GL/ΔUK (IM) 10^4^ HAD	7	1/5
ASFV-G-Δ9GL/ΔUK (IM) 10^4^ HAD	14	5/5
ASFV-G-Δ9GL/ΔUK (IM) 10^4^ HAD	21	4/5
ASFV-G-ΔI177L (IM) 10^2^ HAD	28	20/20
ASFV-G-ΔI177L (IM) 10^6^ HAD	28	5/5
ASFV-G-ΔI177L (O/N) 10^6^ HAD	28	5/5

* IM: intramuscular; ON: oronasal. HAD: Hemadsorption. **^†^** dpv: days post vaccination. **^††^** Clinical observation was performed for 21 days after challenge.

## Data Availability

Not applicable.
